# Socioeconomic disparities in site-specific cancer incidence and mortality: Golestan cohort study

**DOI:** 10.1136/bmjph-2025-003822

**Published:** 2026-06-12

**Authors:** Mahdokht Naghash, Nogol Motamed-Gorji, Nazgol Motamed-Gorji, Gholamreza Roshandel, Hossein Poustchi, Maryam Sharafkhah, Akram Pourshams, Farhad Islami, Masoud Khoshnia, Neal D Freedman, Sanford M Dawsey, Farin Kamangar, Paolo Boffetta, Paul Brennan, Christian C Abnet, Reza Malekzadeh, Arash Etemadi

**Affiliations:** 1 Digestive Oncology Research Center, Digestive Diseases Research Institute, Tehran University of Medical Sciences, Tehran, Iran (the Islamic Republic of); 2 Golestan Research Center of Gastroenterology and Hepatology, Golestan University of Medical Sciences, Gorgan, Iran (the Islamic Republic of); 3 Surveillance, Prevention, and Health Services Research, American Cancer Society, Atlanta, Georgia, USA; 4 Tobacco Control Research Branch, Division of Cancer Control and Population Sciences, National Cancer Institute, National Institutes of Health, Bethesda, Maryland, USA; 5 Metabolic Epidemiology Branch, Division of Cancer Epidemiology and Genetics, National Cancer Institute, NIH, Bethesda, Maryland, USA; 6 Department of Biology, School of Computer, Mathematical and Natural Sciences, Morgan State University, Baltimore, Maryland, USA; 7 Stony Brook Cancer Center, Stony Brook University, Stony Brook, New York, USA; 8 Department of Medical and Surgical Sciences, University of Bologna, Bologna, Italy; 9 International Agency for Research on Cancer, Lyon, France

**Keywords:** Public Health, Epidemiology, Sociodemographic Factors

## Abstract

**Introduction:**

Socioeconomic inequalities are key contributors to cancer disparities. We explored the associations between individual-level socioeconomic status (SES) indicators (composite wealth score, educational attainment and rural/urban residence) and site-specific cancer incidence and mortality.

**Methods:**

For this prospective study, we used data from 49 776 participants of the Golestan Cohort Study in northeastern Iran, enrolled in 2004–2008. Follow-up continued until first primary cancer diagnosis, death, loss to follow-up, or 31 March 2021. We used Cox proportional hazard regression models to estimate HRs and 95% CI, adjusted for several cancer risk factors and all SES indicators.

**Results:**

Over a 14-year median follow-up, the highest wealth score quartile, compared with the lowest, was associated with reduced oesophageal (HR=0.61; 95% CI 0.43 to 0.88) and gastric (HR=0.61; 95% CI 0.43 to 0.86) but elevated breast (HR=2.54; 95% CI 1.32 to 4.89) cancer incidence. The highest education level, compared with none, was associated with reduced oesophageal (HR=0.43; 95% CI 0.24 to 0.79), gastric (HR=0.54; 95% CI 0.35 to 0.84), hepatobiliary (HR=0.33; 95% CI 0.13 to 0.83) and lung (HR=0.44; 95% CI 0.20 to 1.00) cancer incidence. Urban residence was associated with decreased oesophageal (HR=0.63; 95% CI 0.44 to 0.90) but increased colorectal (HR=1.72; 95% CI 1.17 to 2.54), breast (HR=2.37; 95% CI 1.52 to 3.71), hepatobiliary (HR=1.70; 95% CI 1.00 to 2.87) and prostate (HR=2.60; 95% CI 1.24 to 5.45) cancer incidence. Cancer mortality analyses showed similar associations though a few results were not statistically significant due to small sample sizes.

**Conclusions:**

Low wealth score, lack of formal education and rural residence were associated with disparate cancer incidence and mortality, particularly gastrointestinal cancers, in this predominantly rural population from a low- and middle-income country.

**WHAT IS ALREADY KNOWN ON THIS TOPIC**

- Cancer disparities are a significant public health and health equity challenge, attributed largely to differences in social determinants of health. Further research is needed to clarify these disparities, especially in low- and middle-income countries, where current evidence is scarce and resource limitations warrant evidence-based prioritisation of vulnerable populations.

**WHAT THIS STUDY ADDS**

- We found significant associations between socioeconomic status indicators (educational attainment, wealth score and rural vs urban residence) and cancer disparities in a low- and middle-income country, especially for gastrointestinal cancers. Our findings also suggest that the underlying mechanisms of these associations are more complex than those explained by known risk factors such as demographic and lifestyle differences.

**HOW THIS STUDY MIGHT AFFECT RESEARCH, PRACTICE OR POLICY**

- Our findings can inform targeted policies aimed at reducing cancer disparities and highlight the need for further research to identify the mechanisms shaping these disparities.

## Introduction

 Cancer burden is one of the most important public health problems impacted by social, environmental or economic disadvantages, especially in underserved populations. The phenomenon of cancer disparity encompasses disparities in incidence, mortality, survival, survivorship and financial burden, among others.^1 2^ Despite all the progress made in cancer prevention and treatment, cancer disparity remains a major issue across different geographical locations, socioeconomic levels and racial or ethnic groups.^3^ These disparities can be attributed to differences in social determinants of health that lead to disparate risk factor exposure, early detection, accessibility of preventive care and access to proper treatment.^4 5^ Mitigating cancer disparities can alleviate substantial economic burdens as well as facilitate the achievement of health equity.^5^ This requires interventions that are only possible by identifying the contributing socioeconomic factors and their mechanisms of effect.^25^,^7^ While the overall impact is proven, their complex interaction and their influence on the population need further evaluation.^6 8^

While a substantial body of research has explored socioeconomic cancer disparities in high-income countries, evidence from low- and middle-income countries (LMICs) remains notably limited. Most studies from LMICs use area-level data, which introduces ecological fallacy,^9^ and they almost exclusively examine mortality, overlooking the disparities in cancer incidence risk. In addition, many focus on all-cancer trends, potentially masking variations in direction and magnitude of effects on site-specific cancer outcomes.^5^ This evidence gap is particularly crucial given that in LMICs, those with low socioeconomic status (SES) face a double disadvantage, as their vulnerability is compounded by structural barriers, such as inadequate health system infrastructures, limited screening capacity, financial challenges and diagnostic delays.^10 11^ In such resource-limited settings, recognising and prioritising those disproportionately affected by the burden of cancer is essential.

Northeastern Iran is a region with a high risk of upper gastrointestinal cancers,^12^ which have been previously shown to be associated with socioeconomic factors.^13^ The Golestan Cohort Study (GCS), conducted in this region, is a well-established cohort with notable socioeconomic variation, including a high proportion of rural residents and individuals without any formal education. This setting, within an LMIC, is ideally suited for studying the impact of socioeconomic disparities on cancer outcomes, filling the current gap in the literature. In this study, we aimed to evaluate the associations between individual-level SES indicators and site-specific cancer incidence and mortality.

## Materials and methods

GCS is a large population-based prospective study conducted in Golestan province, northeastern Iran. Between 2004 and 2008, in the baseline phase of the study, 50 045 permanent residents of Golestan province between the ages of 40 and 75 were recruited. Urban participants comprised 20% of the study population and were selected by systematic clustering from Gonbad city (the second largest city in the province) based on house numbers. The rural participants (80% of the study population) were selected by population-based sampling from 326 villages. Participation rates were 60% and 75% of the eligible subjects for urban and rural groups, respectively. All GCS participants completed a written informed consent before enrolment.^12^

All GCS participants were interviewed and examined by trained professional healthcare workers. Demographic features, nutritional data, medical and family history, medication history, tobacco and substance use history, and physical activity data were gathered at baseline. Blood pressure and anthropometric measurements were done by the trained interviewers at enrolment. To define the SES, three independent variables in the GCS baseline data were used, including the area of residence (rural or urban), a composite wealth score and educational attainment. The wealth score was computed based on house ownership and size and appliance ownership, using multiple correspondence analysis, as detailed previously,^13^ resulting in identical values for all members of a household. This wealth score was then categorised from quartile 1 (poorest) to quartile 4 (richest). Educational attainment was categorised based on the years of official schooling into three groups: ‘none’, ‘1–5 years’ and ‘6 and more years’. Physical activity was classified as ‘low’, ‘moderate’ and ‘intense’ using the Metabolic Equivalent of Task (in minute/week) tertiles.^14^ Diet quality was categorised into ‘low’, ‘average’ and ‘high’ according to the tertiles of the updated Healthy Eating Index (HEI-2015) score.^15^

### Outcome assessment

During the ongoing follow-up of GCS, the health status of participants is monitored through annual telephone contacts. Cancer incident cases and deaths were identified over a median of 14 years with almost complete follow-up information (>99%). In case of a new cancer diagnosis or death, all available medical documents are gathered, and a verbal autopsy interview is conducted.^16^ The details of the GCS follow-up have been published previously.^16^ Cancer incidence and mortality data gathered during the follow-up were confirmed by linkage to the Golestan population-based cancer registry,^17^ and only cancer cases or deaths confirmed through linkage to the Golestan population-based cancer registry were included in this analysis. All International Classification of Diseases, 10th Revision (ICD-10) codes of C00–C96 were considered as cancer events.^18^ The most common cancer sites were selected for site-specific associations as follows: C15 (oesophagus), C16 (stomach), C18–21 (colon and rectum), C25 (pancreas), C22–24 (liver and biliary tract), C32 (larynx), C34 (lung), C43–44 (melanoma), C50 (breast), C53–56 (cervix, uterus and ovary), C61 (prostate), C64–67 (kidneys and bladder), C71 (brain), C81–88 (lymphomas) and C91–95 (leukaemias). Individuals with a self-reported cancer diagnosis at baseline or earlier were excluded.

### Statistical analysis

We used mean±SD to describe quantitative variables and numbers and percentages for categorical variables. Cancer incidence and mortality rates were calculated as per 100 000 person-years during the follow-up period and age-standardised using the WHO standard world population (ASIR: age-standardised incidence rate and ASMR: age-standardised mortality rate).

The association between SES factors and cancer incidence or mortality was investigated using the Cox proportional hazard regression model. Follow-up continued until first primary cancer diagnosis, death, loss to follow-up or 31 March 2021, whichever happened first. For cancer mortality analyses, cancer diagnosis was replaced by death due to cancer. Overall, analysis included one model for each cancer type diagnosis (incidence models) and each cancer type death (mortality models), for a total of 32 models: two sets of models for 15 cancer types and two models for any cancer event as the main outcome (‘all cancers’ models). The models included all three socioeconomic variables and relevant confounders, consisting of age (10-year age groups), sex (male/female), marital status (married/unmarried), body mass index (BMI) (<25 kg/m^2^, 25–29.9 kg/m^2^ and ≥30 kg/m^2^), physical activity (low/moderate/intense), diet quality (low/average/high), alcohol use (never/ever use), cigarette smoking (never/former/current use) and opium consumption (never/ever use). We further tried including cancer-specific risk factors in the models (hot tea for oesophageal cancer, non-steroidal anti-inflammatory drug use for colorectal cancer, contraceptive pill use and parity for breast and female genital cancers), but they had little impact (<10%) on the point estimates for our main exposures, so they were not part of the final models. All analyses were examined for the proportionality assumption using the Schoenfeld and scaled Schoenfeld residuals, and no violation was observed. A total of 269 participants (0.5%) had missing data on the main study variables and were excluded from the study, and as a result, our analytical sample included 49 776 individuals. Stata Statistical Software V.12 (Stata Corp) was used for data analysis. A p<0.05 was considered statistically significant.

### Patient and public involvement

It was not appropriate or possible to involve patients or the public in the design, or conduct, or reporting, or dissemination plans of our research.

## Results

Table 1 summarises the baseline characteristics of the study population by quartiles of wealth score. Out of the total of 49 776 participants with complete data, 28 645 (57.5%) were female, 39 841 (80%) lived in rural areas and 34 940 (70.2%) had no formal education. Most participants (87.9%) were married and owned their houses (96.6%). As detailed in table 1, individuals in the low wealth score group were more likely to be from rural areas, have no formal education and be unmarried. BMIs of over 25 were more common in the high wealth scores, accompanied by a concomitant increase in diet quality.

**Table 1:** Baseline characteristics of study participants according to their wealth score

Variables		Number (%)				
		Total participants	Wealth score quartiles			
			1 (lowest)	2	3	4 (highest)
Total number		49 776	13 862	11 093	12 524	12 297
Age	35–44 years	10 531 (21.2)	2737 (19.7)	2324 (21.0)	2645 (21.1)	2825 (23.0)
	45–54 years	21 193 (42.6)	5540 (40.0)	4699 (42.4)	5432 (43.4)	5522 (44.9)
	55–64 years	11 954 (24.0)	3402 (24.5)	2672 (24.1)	3078 (24.6)	2802 (22.8)
	65–75 years	6098 (12.3)	2183 (15.8)	1398 (12.6)	1369 (10.9)	1148 (9.3)
Sex	Male	21 131 (42.5)	5697 (41.1)	4541 (40.9)	5372 (42.9)	5521 (44.9)
	Female	28 645 (57.5)	8165 (58.9)	6552 (59.1)	7152 (57.1)	6776 (55.1)
Residence	Urban	9935 (20.0)	893 (6.4)	1342 (12.1)	2448 (19.6)	5252 (42.7)
	Rural	39 841 (80.0)	12 969 (93.6)	9751 (87.9)	10 076 (80.5)	7045 (57.3)
Education	None	34 940 (70.2)	12 120 (87.4)	8758 (79.0)	8543 (68.2)	5519 (44.9)
	≤5 years	8420 (16.9)	1330 (9.6)	1658 (15.0)	2530 (20.2)	2902 (23.6)
	≥6 years	6416 (12.9)	412 (3.0)	677 (6.1)	1451 (11.6)	3876 (31.5)
Marital status*	Married	43 734 (87.9)	11 379 (82.1)	9661 (87.1)	11 254 (89.9)	11 440 (93.0)
	Non-married	5958 (12.0)	2446 (17.7)	1412 (12.7)	1252 (10.0)	848 (6.9)
House ownership	No	1715 (3.5)	535 (3.7)	341 (3.1)	358 (2.9)	481 (3.9)
	Yes	48 061 (96.6)	13 327 (96.1)	10 752 (96.9)	12 166 (97.1)	11 816 (96.1)
Cigarette smoking	Never	41 167 (82.7)	11 270 (81.3)	9258 (83.5)	10 446 (83.4)	10 193 (82.9)
	Former	1703 (3.4)	357 (2.6)	346 (3.1)	440 (3.5)	560 (4.6)
	Current	6906 (13.9)	2235 (16.1)	1489 (13.4)	1638 (13.1)	1544 (12.6)
Opium use	Never	41 347 (83.1)	10 609 (76.5)	9149 (82.5)	10 642 (85.0)	10 947 (89.0)
	Ever	8429 (16.9)	3253 (23.5)	1944 (17.5)	1882 (15.0)	1350 (11.0)
Alcohol use	Never	48 065 (96.6)	13 618 (98.2)	10 844 (97.8)	12 118 (96.8)	11 485 (93.4)
	Ever	1711 (3.4)	244 (1.8)	249 (2.2)	406 (3.2)	812 (6.6)
Ethnicity	Turkmen	37 088 (74.5)	10 847 (78.3)	8057 (72.6)	9598 (76.6)	8586 (69.8)
	Non-Turkmen	12 688 (25.5)	3015 (21.8)	3036 (27.4)	2926 (23.4)	3711 (30.2)
Body mass index*	<25 kg/m^2^	20 227 (40.6)	7500 (54.1)	4990 (45.0)	4525 (36.1)	3212 (26.1)
	25–29.9 kg/m^2^	16 878 (33.9)	3904 (28.2)	3669 (33.1)	4479 (35.8)	4826 (39.3)
	≥30 kg/m^2^	12 663 (25.4)	2455 (17.7)	2434 (21.9)	3516 (28.1)	4258 (34.6)
Physical activity*	Low	16 455 (34.5)	4686 (35.8)	3542 (33.3)	4052 (33.6)	4175 (35.0)
	Average	15 362 (32.2)	3605 (27.6)	3205 (30.2)	3901 (32.3)	4651 (39.0)
	Intense	15 880 (33.3)	4788 (36.6)	3876 (36.5)	4125 (34.2)	3091 (25.9)
Diet quality*	Low	17 614 (36.1)	6529 (48.9)	4702 (43.2)	4255 (34.4)	2128 (17.4)
	Average	16 510 (33.8)	4356 (32.6)	3794 (34.8)	4481 (36.2)	3879 (31.8)
	High	14 681 (30.1)	2463 (18.5)	2394 (22.0)	3630 (29.4)	6194 (50.8)

* Data were missing for marital status in 84, body mass index in 8, physical activity in 2079 and diet quality in 971 individuals.

Over a median follow-up period of 14 years (equivalent to 655 917.4 person-years), a total of 2183 incident cancer cases occurred (ASIR: 247.2 per 100 000 person-years), and there were 1607 fatal cancer cases (ASMR: 181.05 per 100 000 person-years). Both overall cancer ASIR and ASMR were lower in the highest wealth scores compared with the lowest wealth scores (online supplemental table S1). Tables2  3 show the crude and adjusted incidence and mortality rates and mortality-to-incidence ratios for each of the most common cancer types in our study by wealth score quartiles. The highest adjusted incidence and mortality rates were for oesophageal and gastric cancers, followed by colorectal and lung cancers. For both oesophageal and gastric cancers, individuals in the lowest wealth score quartile had the highest ASIRs and ASMRs, while the inverse was true for colorectal and breast cancers.

**Table 2:** Crude and standardised* rates of gastrointestinal cancer incidence, mortality and mortality-to-incidence ratio per 100 000 person-years

			Oesophagus	Stomach	Colorectal	Liver and gallbladder	Pancreas
Cancer incidence	Total number		394	384	161	101	94
	Crude rates		60.1	58.5	24.5	15.4	14.3
	Age-standardised rates		43.6	43.7	18.4	11.1	11.2
	Age-standardised rates by wealth score quartiles	1	62.3	58.8	13.3	14.5	10.7
		2	51.7	45.6	9.4	7.5	8.5
		3	35.7	42	18.8	11.5	15.8
		4	24.2	27.5	31.3	10	9.5
Cancer mortality	Total number		326	333	94	85	89
	Crude rates		49.4	50.4	14.2	12.9	13.5
	Age-standardised rates		36.9	39.1	11	9.9	9.7
	Age-standardised rates by wealth score quartiles	1	55.5	51.4	8.6	12.7	10.6
		2	44.7	40.7	6	6.2	8
		3	26.8	34.6	13.1	8.9	10.8
		4	20.1	29.1	15.7	11.1	9.1
Cancer mortality-to-incidence†	Age-standardised rate ratio		0.8	0.9	0.6	0.9	0.9
	Age-standardised rate ratio by wealth score quartiles	1	0.9	0.9	0.6	0.9	1
		2	0.9	0.9	0.6	0.8	0.9
		3	0.8	0.8	0.7	0.8	0.7
		4	0.8	1.1	0.5	1.1	1

* Direct standardised using the WHO standard world population.

† Numbers above 1 indicate cases who were only identified after death.

**Table 3:** Crude and standardised* rates of non-gastrointestinal cancer incidence, mortality and mortality-to-incidence ratio per 100 000 person-years

			Lung	Breast	Female genital	Kidney and bladder	Leukaemia	Lymphoma
Cancer incidence	Total number		119	112	104	83	74	73
	Crude rates		18.1	17.1	15.9	12.7	11.3	11.1
	Age-standardised rates		12.5	15.8	11	8.7	8.2	7.5
	Age-standardised rates by wealth score quartiles	1	15.5	9.4	10.6	8.2	8.2	8.4
		2	10.7	14.3	10.4	9	8.4	6.4
		3	12	12.2	13.9	9.9	8.4	9.2
		4	11.5	27.4	9	7.7	7.9	6
Cancer mortality	Total number		106	33	64	49	56	50
	Crude rates		16	5	9.7	7.4	8.5	7.6
	Age-standardised rates		11.1	4.4	6.4	5.2	6.3	5
	Age-standardised rates by wealth score quartiles	1	13.2	4.4	8.1	5.9	6.1	5
		2	9.2	3.5	7.5	4.5	7.2	4.5
		3	11.2	2.6	6.5	6	7.1	7.7
		4	10.5	6.9	3.7	4.4	5	2.7
Cancer mortality-to-incidence	Age-standardised rate ratio		0.9	0.3	0.6	0.6	0.8	0.7
	Age-standardised rate ratio by wealth score quartiles	1	0.9	0.5	0.8	0.7	0.7	0.6
		2	0.9	0.2	0.7	0.5	0.9	0.7
		3	0.9	0.2	0.5	0.6	0.8	0.8
		4	0.9	0.3	0.4	0.6	0.6	0.5

* Direct standardised using the WHO standard world population.

**Figure 1:**
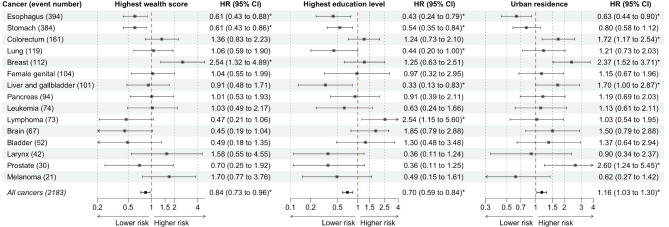
HRs and 95% CIs of site-specific cancer incidence by socioeconomic factors. The highest quartile of wealth score was compared with the lowest, having 6 or more years of education was compared with no formal education, and urban residence was compared with rural. The models included all three socioeconomic variables and age, sex, marital status, BMI, physical activity, diet quality, alcohol use, cigarette smoking and opium consumption. ‘All cancers’ models also include less common cancer sites not listed here. The x-axis is logarithmic scale. *p<0.05. BMI, body mass index.

Figures1  2 show the adjusted HRs and CIs for the associations between SES factors and the incidence and mortality of common cancer types. Compared with the first quartile of wealth score, individuals in the highest wealth score quartile had nearly 40% lower risk of oesophageal cancer incidence (HR=0.61; 95% CI 0.43 to 0.88). This inverse association was also seen for oesophageal cancer mortality (HR=0.52; 95% CI 0.35 to 0.79). Being in the highest wealth score quartile was also associated with lower incidence and mortality for gastric cancer with HRs of 0.61 (95% CI 0.43 to 0.86) and 0.64 (95% CI 0.44 to 0.93), respectively. The highest wealth score quartile was associated with a higher incidence of breast cancer, with an HR of 2.54 (95% CI 1.32 to 4.89). The highest wealth score was also associated with a significantly reduced risk of bladder cancer mortality (HR=0.16; 95% CI 0.03 to 0.87). Details of the associations between quartiles of wealth score and site-specific cancer incidence and mortality are presented in online supplemental table S2.

**Figure 2:**
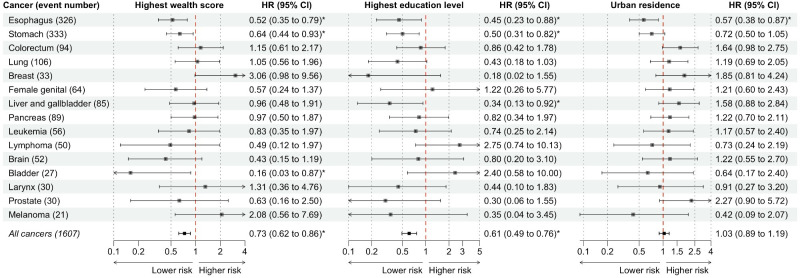
HRs and 95% CIs of site-specific cancer mortality by socioeconomic factors. The highest quartile of wealth score was compared with the lowest, having 6 or more years of education was compared with no formal education, and urban residence was compared with rural. The models included all three socioeconomic variables and age, sex, marital status, BMI, physical activity, diet quality, alcohol use, cigarette smoking, and opium consumption. ‘All cancers’ models also include less common cancer sites not listed here. The x-axis is logarithmic scale. *p<0.05. BMI, body mass index.

In comparison to no formal education, having 6 years or more of formal education was associated with reduced incidence (HR=0.43; 95% CI 0.24 to 0.79) and mortality of oesophageal cancer (HR=0.45; 95% CI 0.23 to 0.88). Those with over 6 years of education also had a decreased incidence and mortality of gastric cancer with HRs of 0.54 (95% CI 0.35 to 0.84) and 0.50 (95% CI 0.31 to 0.82), respectively. The highest level of educational attainment was associated with reduced incidence of lung cancer (HR=0.44; 95% CI 0.20 to 1.00), and both reduced incidence (HR=0.33; 95% CI 0.13 to 0.83) and mortality (HR=0.34; 95% CI 0.13 to 0.92) of hepatobiliary cancers. We observed an increased risk of lymphoma incidence among individuals with more than 6 years of formal education (HR=2.54; 95% CI 1.15 to 5.60). Details of the associations between educational attainment and site-specific cancer incidence and mortality are presented in online supplemental table S2.

Living in urban areas was associated with decreased risk of both oesophageal cancer incidence (HR=0.63; 95% CI 0.44 to 0.90) and mortality (HR=0.57; 95% CI 0.38 to 0.87). On the contrary, urban residents were at a higher risk of incident colorectal cancer (HR=1.72; 95% CI 1.17 to 2.54), breast cancer (HR=2.37; 95% CI 1.52 to 3.71), liver and gallbladder cancer (HR=1.70; 95% CI 1.00 to 2.87) and prostate cancer (HR=2.60; 95% CI 1.24 to 5.45).

## Discussion

We found significant associations between individual-level SES indicators and cancer incidence and mortality in Golestan in northeastern Iran, a low- and middle-income country. Cancer ASIR and ASMR were lower in the highest wealth scores compared with the lowest. The upper gastrointestinal cancers (oesophagus and stomach), as the most common incident cancers in this population, showed consistent associations with all studied socioeconomic factors. A high wealth score was associated with reductions in the risk of upper gastrointestinal cancers and an increased risk of breast cancer incidence. A high level of educational attainment was also associated with a decreased risk of upper gastrointestinal, lung, and hepatobiliary cancers and lymphoma. Living in urban areas was associated with reduced risk of upper gastrointestinal cancers, especially oesophageal cancer, and increased risk of colorectal, breast, hepatobiliary and prostate cancers. Cancer mortality analyses showed similar associations, though a few results were not statistically significant due to small sample sizes

Wealth and educational attainment tend to improve access to healthcare, promote awareness about healthy lifestyles, increase receptiveness to health education and therefore enhance early diagnosis, risk factor prevention and treatment quality. Additionally, both these factors have an inherent interplay with occupation and housing status that can have health impacts through harmful exposures.^5^ In our study, these SES indicators were inversely associated with the incidence and mortality of upper gastrointestinal cancers, independent of each other and several known risk factors. Similar findings were observed for gastric cancer in a study conducted within the European Prospective Investigation into Cancer and Nutrition cohort, where established risk factors of gastric cancer, similar to those we adjusted for, only partially explained the observed socioeconomic gradient.^19^ Studies from Sweden, the USA, China, France and Canada have also reported higher incidence and mortality rates of upper gastrointestinal cancers among individuals with lower income or educational attainment.^20^,^24^ A prior study in Golestan has found declining trends of oesophageal and gastric cancers, attributed to reduced exposure to SES-related risk factors across consecutive cohorts. Despite this declining trend, ASIR and ASMR of both oesophageal and gastric cancer in the area are still among the highest in the world.^25^

In addition to upper gastrointestinal cancers, educational attainment was inversely associated with the incidence of lung cancer and the incidence and mortality of hepatobiliary cancers. Analysis of SEER data (Surveillance, Epidemiology and End Results) has also shown higher lung cancer incidence among less educated individuals.^26^ Reduced risk of hepatobiliary cancers in individuals with advanced education has also been present in studies done in Europe, China and the USA.^21 22 27^ Given that in our study, these educational gradients persisted after adjusting for some of the prominent risk factors, including smoking and alcohol use, the association appears to be driven by unmeasured environmental, structural, social or lifestyle factors. The increase we observed in the risk of breast cancer incidence in higher wealth scores has also been observed in many studies.^20 22 23 28^ Reproductive factors (ie, low parity, high age at first birth, low age at menarche) and hormone replacement therapy may play a key role in increased risk among wealthy women.^29^

There was a large disparity in how rural/urban residence affected various cancer risks in our study. Our findings indicated that rural residents had higher incidence and mortality rates of upper gastrointestinal cancers (especially oesophageal cancer), which are the most common cancers in our study population.^25^ This is consistent with previous findings from China, another high-risk area for oesophageal cancer,^30^ and data from SEER and the North American Association of Central Cancer Registries.^31 32^ Similar to other rural areas with a high incidence of oesophageal cancer,^33 34^ rural residents of Golestan are exposed to polycyclic aromatic hydrocarbons at higher levels compared with urban residents,^35^ which can partially explain the observed disparity. While *Helicobacter pylori* infection is the most important cause of gastric cancer,^36^ the prevalence of anti-Hp and anti-cagA antibodies among healthy individuals in Golestan province is not significantly different between rural and urban residents,^37^ so this is unlikely to explain the difference we report here.

We observed an increased risk of colorectal cancer incidence in urban areas. While the global burden of colorectal cancer has been decreasing in high-incidence regions such as North America and Australia, presumably because of the widespread use of colorectal cancer screening, it has been on the rise in LMICs in East Asia and North Africa and the Middle East, where the incidence of these cancers has traditionally been relatively low.^38 39^ These changes in LMICs have been attributed in part to the expansion of the Western lifestyle and urbanisation.^40^ Our study also provided further evidence for the increased risk of breast and prostate cancers in urban populations, a finding well-documented in the literature. A meta-analysis of 31 studies has also indicated that urban residence is associated with an increase in breast cancer incidence, proposing reproductive factors, diet and physical activity as other potential explanations.^41^ Previous research in Golestan observed higher rates of breast cancer incidence in urban areas, with an increasing trend since 2004. The authors showed a birth cohort effect attributable to reproductive factors such as fertility rate decline, late marriage age and reduced breastfeeding rates.^42^ Our findings about prostate cancer also corroborate the findings of a systematic review that found a higher incidence of prostate cancer among urban men.^43^ Given that colorectal, breast and prostate cancers are detectable through screening, these disparities may reflect the inequalities in access to opportunistic screening in Iran, where no organised screening programmes exist, and rural residents have limited access to diagnostic services.^44^

We used a well-established cohort in an underrepresented population, within a region which is part of the so-called ‘Asian oesophageal cancer belt’, and one of the highest rates of upper gastrointestinal cancers in the world. Our data also included detailed, comprehensive data on SES factors with high proportions of rural residents and low levels of formal education, making it an appropriate sample for studying the socioeconomic correlates of cancer disparity. The long follow-up and negligible loss during this period are among the other strengths of the current study. Our study had a number of limitations. We conducted adjustments for many general and site-specific risk factors and adjusted each model for the other two SES indicators to further clarify the associations. Even so, we could not control for multiple other potential confounding factors such as full reproductive factors profile, hepatitis and *H. pylori* infection. Our study only provides information related to one province, where cultural differences and habits may affect cancer outcomes. For example, in this province, alcohol consumption is very uncommon, and this may impact generalisation to populations where alcohol use is common. We didn’t collect direct reports of income, as these reports can be inaccurate and hard to gather,^45^ and the wealth score method has been tested in this population.^13^

## Conclusions

In this prospective cohort study, we found differences in site-specific cancer incidence and mortality rates by SES in a predominantly rural population from a low-middle-income country. These associations remained after the adjustment for some prominent risk factors, suggesting intricate pathways for the effects of social determinants of health on cancer risk beyond the observed demographic and lifestyle differences. Oesophageal and gastric cancers were especially more common in individuals with low wealth scores, low educational attainment and those living in rural areas. The incidence and mortality of other cancers were also affected by socioeconomic factors, especially educational attainment. This study further builds on the importance of studying the complex interplay between cancer disparities and SES to recognise and address the underlying contributing factors.

## Supplementary material

10.1136/bmjph-2025-003822 online supplemental file 1

## Data Availability

Data are available on reasonable request.
