# Supplementary material for: Socioeconomic disparities in site-specific cancer incidence and mortality: Golestan cohort study
Source: BMJ Public Health. 2026 Jun 12;4(2):e003822. doi: 10.1136/bmjph-2025-003822 (PMC13289312; doi:10.1136/bmjph-2025-003822)
Supplement: online supplemental file 1 [file bmjph-4-2-s001.docx]

| Supplementary Table-S1: Overall cancer Incidence and mortality rates according to wealth score quartiles | | | | | | |
| --- | --- | --- | --- | --- | --- | --- |
| Variables | | Total participants | Wealth score quartiles | | | |
|  |  |  | 1st | 2nd | 3rd | 4th |
| Total number | | 49,776 | 13,862 | 11,093 | 12,524 | 12,297 |
| Cancer incidence | Number (%) | 2183 (4.4) | 708 (5.1) | 470 (4.2) | 551 (4.4) | 454 (3.7) |
|  | Person-Year | 655926.21 | 179000.67 | 145128.18 | 164710.95 | 167086.42 |
|  | CIR^a^ | 332.8 | 395.5 | 323.8 | 334.5 | 271.7 |
|  | ASIR^a^ | 247.2 | 277.5 | 242.5 | 248.7 | 217.5 |
|  | Mean age | 63.50  (63.09-63.90) | 63.71  (63.00-64.42) | 63.96  (63.08-64.84) | 63.60  (62.80-64.39) | 62.57  (61.67-63.47) |
| Cancer mortality | Number (%) | 1607 (3.2) | 572 (4.1) | 365 (3.3) | 385 (3.1) | 285 (2.3) |
|  | Person-Year | 660581.58 | 180086.16 | 146048.33 | 166019.53 | 168427.56 |
|  | CMR^a^ | 243.3 | 317.6 | 249.9 | 231.9 | 169.2 |
|  | ASMR^a^ | 181.1 | 220.7 | 180.10 | 179.20 | 141.30 |
|  | Mean age | 65.20  (64.74-65.67) | 64.99  (64.21-65.78) | 65.33  (64.38-66.28) | 65.59  (64.62-66.56) | 64.93  (63.82-66.05) |

^a^ per 100,000 person-years

CIR: crude incidence rate / ASIR: age-standardized incidence rate

CMR: crude mortality rate / ASMR: age-standardized mortality rate

| Supplementary Table-S2 – Hazard ratios and 95% CIs of incidence and mortality of site-specific cancers for quartiles of wealth score and levels of educational attainment | | | | | | | | | | | | | |
| --- | --- | --- | --- | --- | --- | --- | --- | --- | --- | --- | --- | --- | --- |
|  |  | Quartiles of wealth score | | | | | |  | Formal education | | | | |
|  | N | 1 (lowest) | 2 | 3 | 4 (highest) | p-for-trend |  | | | None | ≤ 5 years | ≥ 6 years | p-for-trend |
| Cancer incidence |  |  |  |  |  |  |  | | |  |  |  |  |
| Esophagus | 394 | Reference | 0.88 (0.69-1.14) | 0.77 (0.59-1.01) | 0.61 (0.43-0.88)* | 0.005* |  | | | Reference | 0.87 (0.62-1.21) | 0.43 (0.24-0.79)* | 0.008* |
| Stomach | 384 | Reference | 0.78 (0.60-1.02) | 0.82 (0.62-1.07) | 0.61 (0.43-0.86)* | 0.007* |  | | | Reference | 0.55 (0.39-0.77)* | 0.54 (0.35-0.84)* | <0.001* |
| Colorectal | 161 | Reference | 0.66 (0.38-1.14) | 1.05 (0.66-1.69) | 1.36 (0.83-2.23) | 0.112 |  | | | Reference | 1.00 (0.63-1.60) | 1.24 (0.73-2.10) | 0.368 |
| Lung | 119 | Reference | 0.81 (0.48-1.39) | 0.93 (0.55-1.57) | 1.06 (0.59-1.90) | 0.1 |  | | | Reference | 1.43 (0.88-2.33) | 0.44 (0.20-1.00)* | 0.04* |
| Breast | 112 | Reference | 1.35 (0.68-2.70) | 1.91 (1.01-3.61)* | 2.54 (1.32-4.89)* | 0.002* |  | | | Reference | 1.25 (0.74-2.13) | 1.25 (0.63-2.51) | 0.428 |
| Female genital | 104 | Reference | 1.08 (0.60-1.93) | 1.27 (0.73-2.22) | 1.04 (0.55-1.99) | 0.4 |  | | | Reference | 0.79 (0.36-1.71) | 0.97 (0.32-2.95) | 0.1 |
| Liver and gallbladder | 101 | Reference | 0.62 (0.33-1.15) | 0.83 (0.47-1.46) | 0.91 (0.48-1.71) | 0.2 |  | | | Reference | 0.70 (0.38-1.29) | 0.33 (0.13-0.83)* | 0.05* |
| Pancreas | 94 | Reference | 0.74 (0.40-1.38) | 1.18 (0.68-2.03) | 1.01 (0.53-1.93) | 0.655 |  | | | Reference | 1.17 (0.63-2.15) | 0.91 (0.39-2.11) | 0.961 |
| Kidney and bladder | 83 | Reference | 1.03 (0.53-2.00) | 1.11 (0.59-2.10) | 0.74 (0.35-1.60) | 0.7 |  | | | Reference | 1.03 (0.56-1.90) | 0.68 (0.30-1.54) | 0.1 |
| Leukemia | 74 | Reference | 1.08 (0.56-2.06) | 1.10 (0.58-2.09) | 1.03 (0.49-2.17) | 0.875 |  | | | Reference | 0.87 (0.42-1.80) | 0.63 (0.24-1.66) | 0.343 |
| Lymphoma | 73 | Reference | 0.73 (0.37-1.44) | 0.97 (0.52-1.80) | 0.47 (0.21-1.06) | 0.177 |  | | | Reference | 1.84 (0.95-3.56) | 2.54 (1.15-5.60)* | 0.021* |
| Melanoma | 60 | Reference | 0.57 (0.21-1.56) | 1.06 (0.46-2.43) | 0.70 (0.25-1.92) | 0.6 |  | | | Reference | 0.75 (0.32-1.72) | 0.49 (0.15-1.61) | 0.2 |
| Prostate | 46 | Reference | 0.85 (0.38-1.89) | 1.27 (0.61-2.62) | 1.70 (0.77-3.76) | 0.06 |  | | | Reference | 1.23 (0.58-2.60) | 0.36 (0.11-1.25) | 0.08 |
| Larynx | 42 | Reference | 1.63 (0.68-3.87) | 1.31 (0.51-3.33) | 1.58 (0.55-4.55) | 0.3 |  | | | Reference | 0.69 (0.30-1.64) | 0.36 (0.11-1.24) | 0.1 |
| Cancer mortality |  |  |  |  |  |  |  | | |  |  |  |  |
| Esophagus | 326 | Reference | 0.88 (0.68-1.15) | 0.63 (0.46-0.86)* | 0.52 (0.35-0.79)* | <0.001* |  | | | Reference | 0.92 (0.64-1.33) | 0.45 (0.23-0.88)* | 0.037* |
| Stomach | 333 | Reference | 0.77 (0.58-1.03) | 0.75 (0.56-1.01) | 0.64 (0.44-0.93)* | 0.01* |  | | | Reference | 0.56 (0.39-0.81)* | 0.50 (0.31-0.82)* | <0.001* |
| Colorectum | 94 | Reference | 0.71 (0.36-1.38) | 0.90 (0.49-1.64) | 1.15 (0.61-2.17) | 0.58 |  | | | Reference | 1.01 (0.56-1.84) | 0.86 (0.42-1.78) | 0.813 |
| Lung | 106 | Reference | 0.80 (0.45-1.43) | 0.96 (0.55-1.66) | 1.05 (0.56-1.96) | 0.4 |  | | | Reference | 1.40 (0.83-2.34) | 0.43 (0.18-1.03) | 0.19 |
| Breast | 33 | Reference | 1.91 (0.62-5.88) | 1.31 (0.39-4.41) | 3.06 (0.98-9.56) | 0.08 |  | | | Reference | 0.74 (0.26-2.09) | 0.18 (0.02-1.55) | 0.127 |
| Female genital | 64 | Reference | 0.94 (0.48-1.87) | 0.90 (0.45-1.81) | 0.57 (0.24-1.37) | 0.7 |  | | | Reference | 1.08 (0.40-2.91) | 1.22 (0.26-5.77) | 0.2 |
| Liver and gallbladder | 85 | Reference | 0.59 (0.30-1.18) | 0.78 (0.42-1.46) | 0.96 (0.48-1.91) | 0.1 |  | | | Reference | 0.62 (0.31-1.27) | 0.34 (0.13-0.92)* | 0.05* |
| Pancreas | 89 | Reference | 0.70 (0.37-1.31) | 1.05 (0.60-1.85) | 0.97 (0.50-1.87) | 0.837 |  | | | Reference | 1.22 (0.66-2.26) | 0.82 (0.34-1.97) | 0.876 |
| Kidney and bladder | 49 | Reference | 0.82 (0.31-2.01) | 0.84 (0.33-1.93) | 0.46 (0.13-1.67) | 0.2 |  | | | Reference | 1.45 (0.77-2.67) | 0.77 (0.29-2.46) | 0.3 |
| Leukemia | 56 | Reference | 1.18 (0.59-2.39) | 0.84 (0.39-1.81) | 0.83 (0.35-1.97) | 0.54 |  | | | Reference | 0.75 (0.31-1.82) | 0.74 (0.25-2.14) | 0.49 |
| Lymphoma | 50 | Reference | 1.01 (0.33-3.03) | 1.54 (0.57-4.17) | 0.49 (0.12-1.97) | 0.68 |  | | | Reference | 2.02 (0.70-5.90) | 2.75 (0.74-10.13) | 0.149 |
| Melanoma | 21 | Reference | 0.99 (0.28-3.52) | 1.05 (0.29-3.84) | 2.08 (0.56-7.69) | 0.09 |  | | | Reference | 0.54 (0.11-2.71) | 0.35 (0.04-3.45) | 0.6 |
| Prostate | 30 | Reference | 0.68 (0.19-2.43) | 1.68 (0.60-4.69) | 0.63 (0.16-2.50) | 0.1 |  | | | Reference | 1.06 (0.42-2.69) | 0.30 (0.06-1.55) | 0.2 |
| Larynx | 30 | Reference | 1.10 (0.38-3.21) | 1.31 (0.46-3.75) | 1.31 (0.36-4.76) | 0.1 |  | | | Reference | 0.37 (0.10-1.33) | 0.44 (0.10-1.83) | 0.8 |

**P* < 0.05
